# Supplementary material for: Identification of Tumor-Suppressive miR-30e-3p Targets: Involvement of SERPINE1 in the Molecular Pathogenesis of Head and Neck Squamous Cell Carcinoma
Source: Int J Mol Sci. 2022 Mar 30;23(7):3808. doi: 10.3390/ijms23073808 (PMC8998321; doi:10.3390/ijms23073808)
Supplement: Supplementary file 1 [file ijms-23-03808-s001.zip › Supplemental Figure.pptx]

## Slide 1
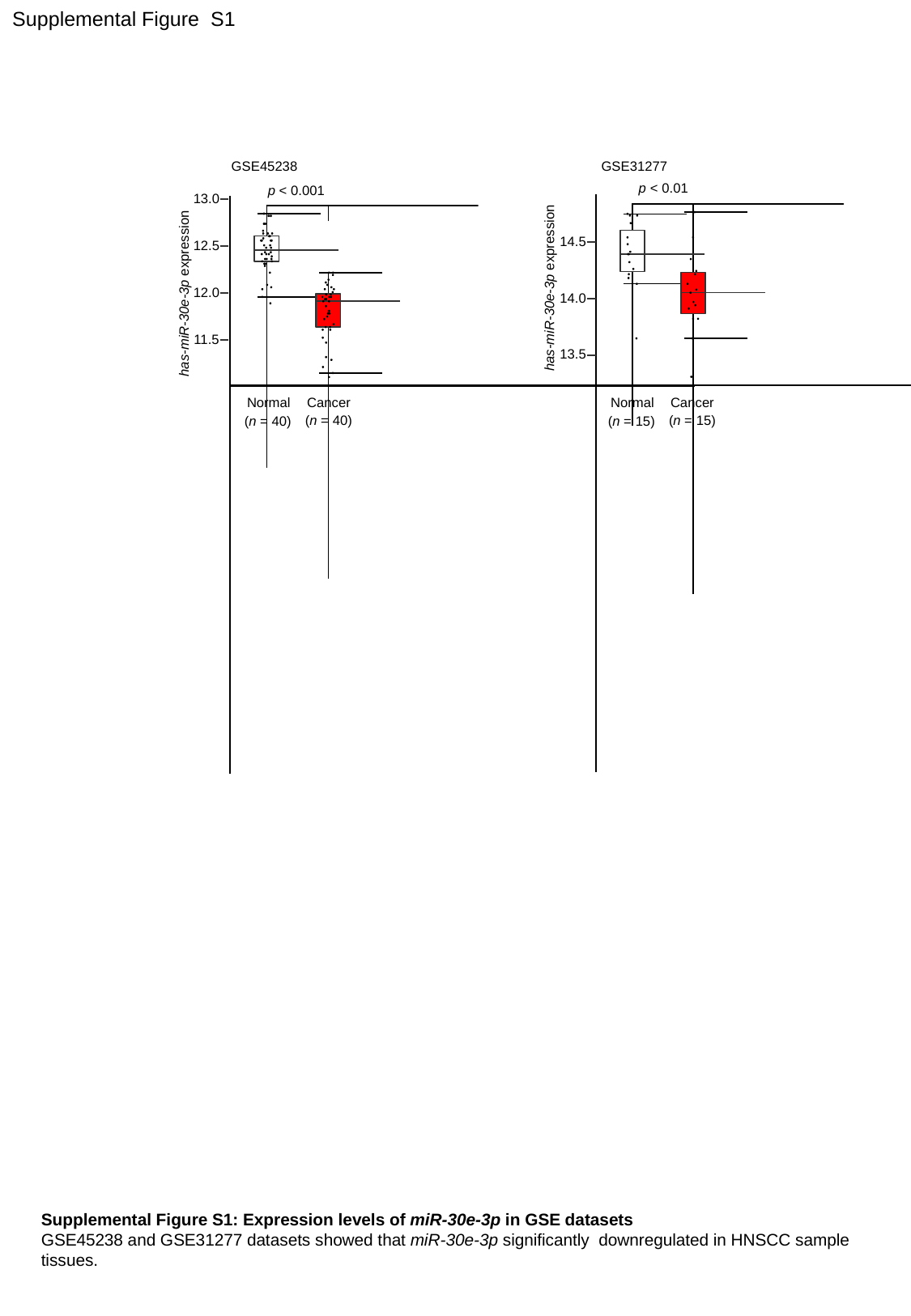

Supplemental Figure S1
GSE45238
GSE31277
p < 0.01
p < 0.001
13.0
12.5
has-miR-30e-3p expression
12.0
11.5
Normal
Cancer
(n = 40)
(n = 40)
14.5
has-miR-30e-3p expression
14.0
13.5
Normal
Cancer
(n = 15)
(n = 15)
Supplemental Figure S1: Expression levels of miR-30e-3p in GSE datasets
GSE45238 and GSE31277 datasets showed that miR-30e-3p significantly downregulated in HNSCC sample tissues.

## Slide 2
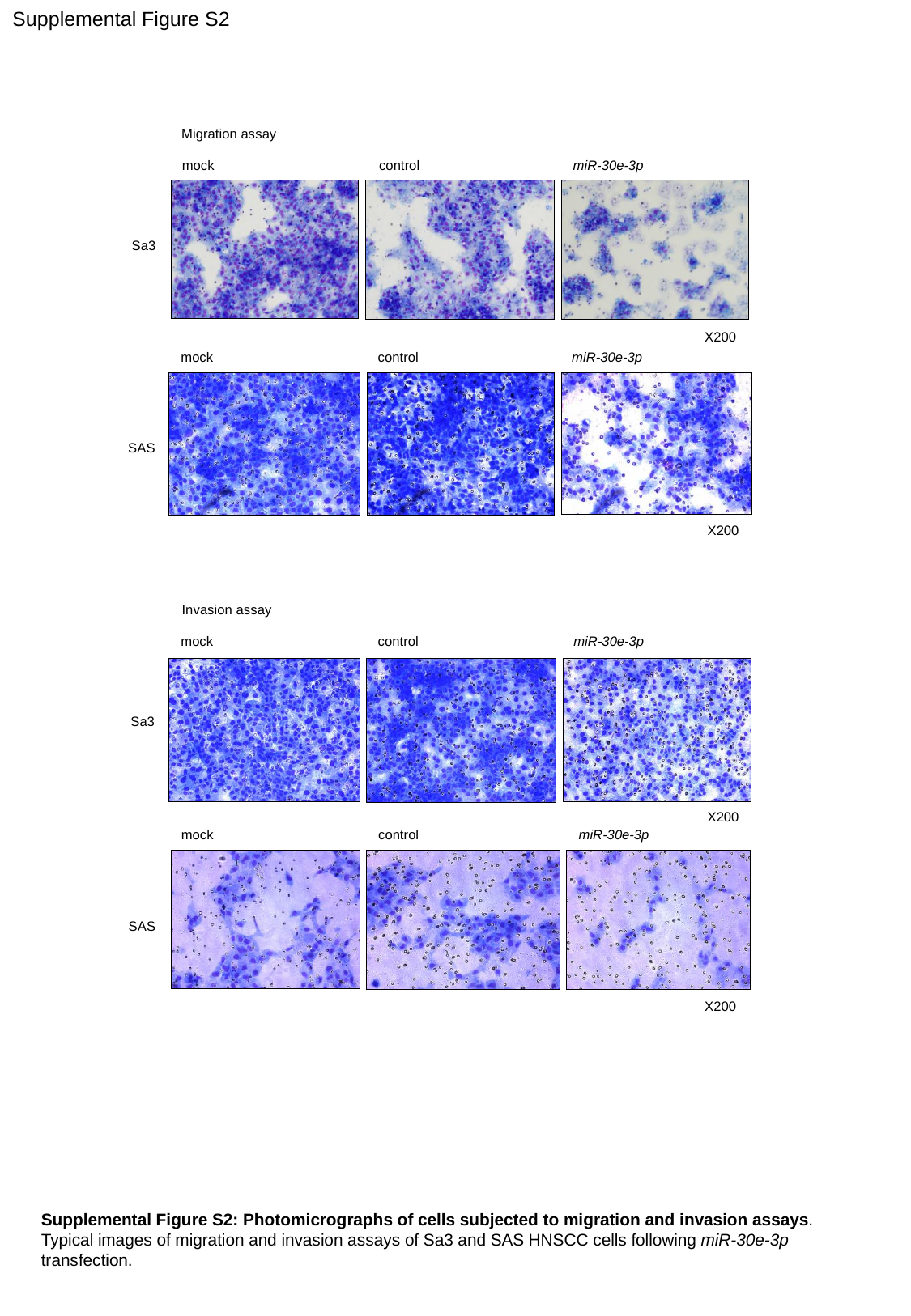

Supplemental Figure S2
Migration assay
mock
control
miR-30e-3p
Sa3
mock
control
miR-30e-3p
SAS
X200
X200
Invasion assay
mock
control
miR-30e-3p
Sa3
X200
mock
control
miR-30e-3p
SAS
X200
Supplemental Figure S2: Photomicrographs of cells subjected to migration and invasion assays.
Typical images of migration and invasion assays of Sa3 and SAS HNSCC cells following miR-30e-3p transfection.

## Slide 3
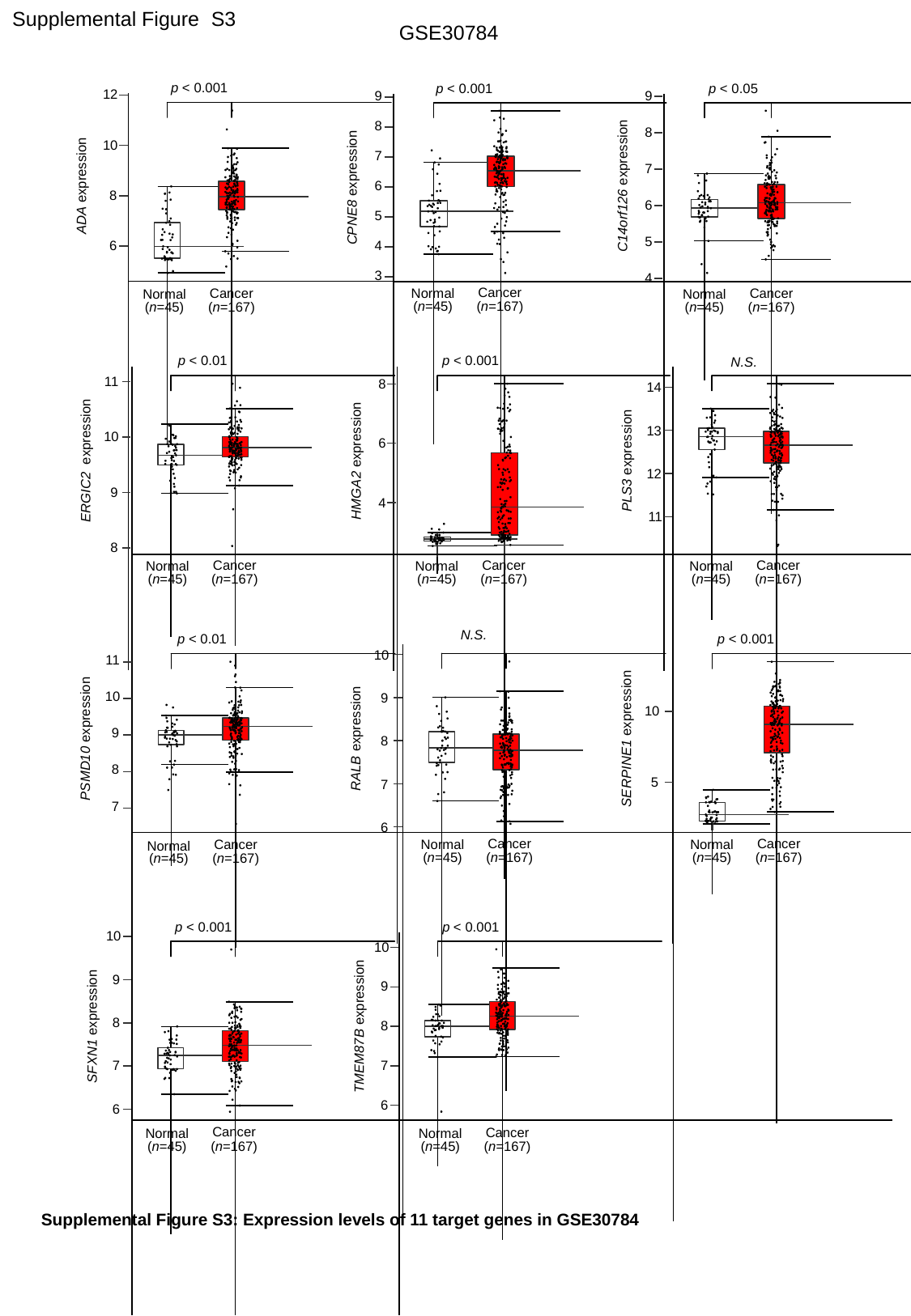

Supplemental Figure S3
GSE30784
p < 0.001
p < 0.001
p < 0.05
12
9
9
8
8
10
7
7
 ADA expression
 C14orf126 expression
CPNE8 expression
6
8
6
5
5
6
4
3
4
Normal
Cancer
(n=45)
(n=167)
Normal
Cancer
(n=45)
(n=167)
Normal
Cancer
(n=45)
(n=167)
p < 0.001
p < 0.01
N.S.
11
8
14
13
10
6
HMGA2 expression
ERGIC2 expression
PLS3 expression
12
9
4
11
8
Normal
Cancer
(n=45)
(n=167)
Normal
Cancer
(n=45)
(n=167)
Normal
Cancer
(n=45)
(n=167)
p < 0.01
p < 0.001
N.S.
10
11
10
9
10
9
RALB expression
SERPINE1 expression
PSMD10 expression
8
8
5
7
7
6
Normal
Cancer
(n=45)
(n=167)
Normal
Cancer
(n=45)
(n=167)
Normal
Cancer
(n=45)
(n=167)
p < 0.001
p < 0.001
10
10
9
9
TMEM87B expression
SFXN1 expression
8
8
7
7
6
6
Normal
Cancer
(n=45)
(n=167)
Normal
Cancer
(n=45)
(n=167)
Supplemental Figure S3: Expression levels of 11 target genes in GSE30784

## Slide 4
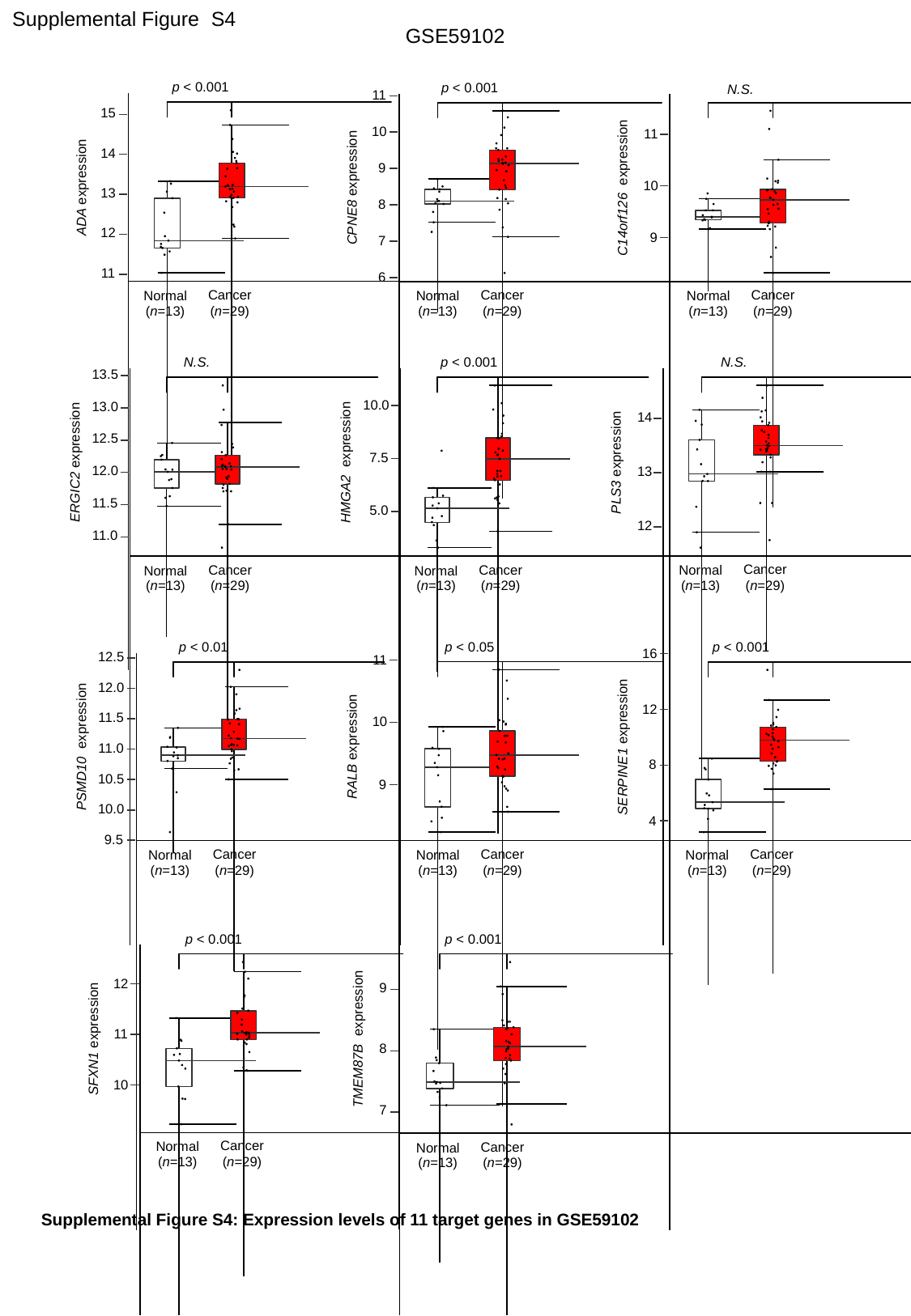

Supplemental Figure S4
GSE59102
p < 0.001
p < 0.001
N.S.
11
15
10
11
14
9
ADA expression
C14orf126 expression
CPNE8 expression
10
13
8
12
9
7
11
6
Normal
Cancer
(n=13)
(n=29)
Normal
Cancer
(n=13)
(n=29)
Normal
Cancer
(n=13)
(n=29)
p < 0.001
N.S.
N.S.
13.5
10.0
13.0
14
12.5
HMGA2 expression
PLS3 expression
ERGIC2 expression
7.5
12.0
13
11.5
5.0
12
11.0
Normal
Cancer
(n=13)
(n=29)
Normal
Cancer
(n=13)
(n=29)
Normal
Cancer
(n=13)
(n=29)
p < 0.01
p < 0.05
p < 0.001
16
12.5
11
12.0
12
11.5
10
RALB expression
PSMD10 expression
SERPINE1 expression
11.0
8
10.5
9
10.0
4
9.5
Normal
Cancer
(n=13)
(n=29)
Normal
Cancer
(n=13)
(n=29)
Normal
Cancer
(n=13)
(n=29)
p < 0.001
p < 0.001
12
9
SFXN1 expression
TMEM87B expression
11
8
10
7
Normal
Cancer
(n=13)
(n=29)
Normal
Cancer
(n=13)
(n=29)
Supplemental Figure S4: Expression levels of 11 target genes in GSE59102

## Slide 5
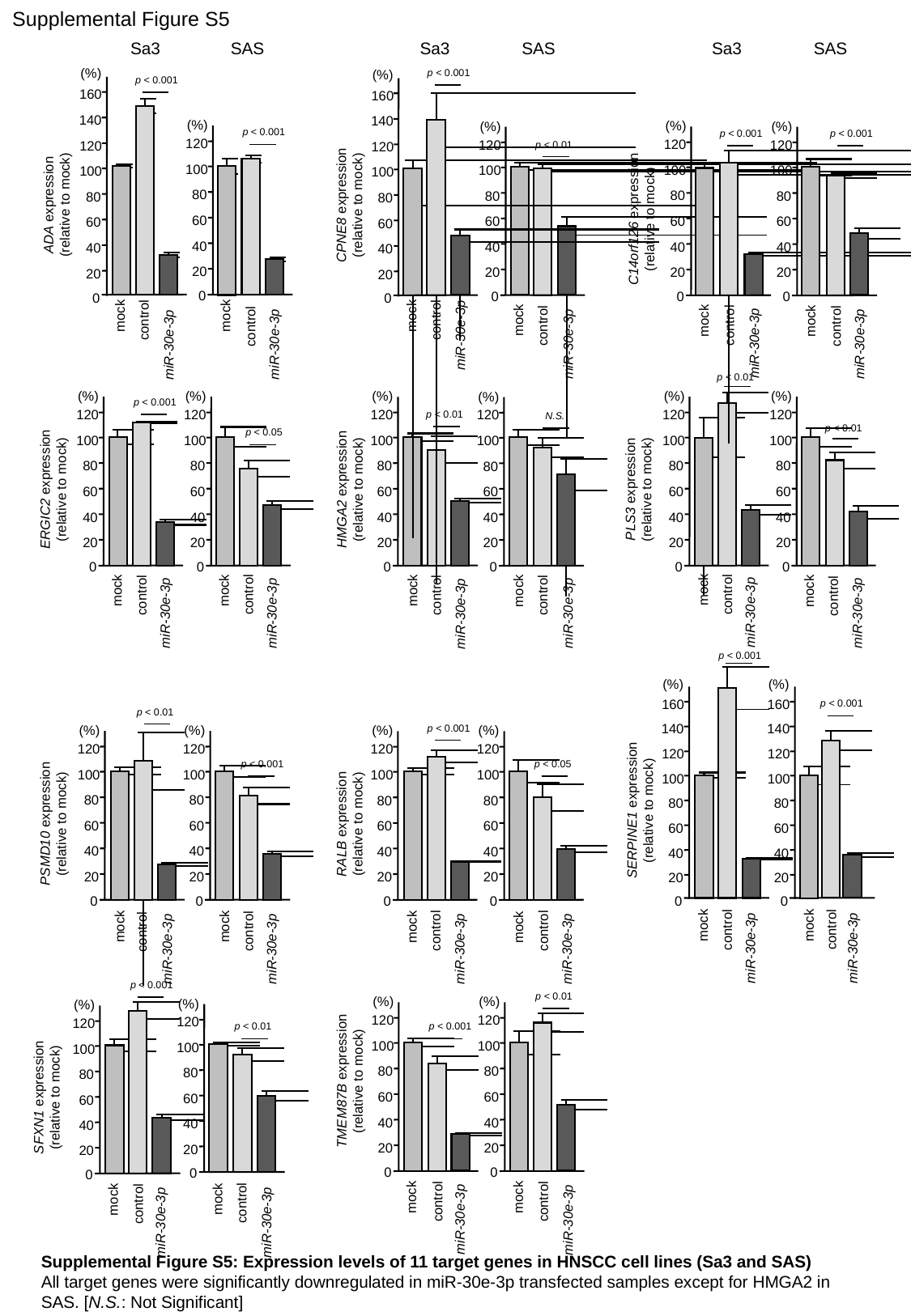

Supplemental Figure S5
Sa3
SAS
Sa3
SAS
Sa3
SAS
p < 0.001
(%)
160
140
120
100
ADA expression
(relative to mock)
80
60
40
20
0
mock
control
miR-30e-3p
(%)
120
100
80
60
40
20
0
mock
control
miR-30e-3p
(%)
160
140
120
100
CPNE8 expression
(relative to mock)
80
60
40
20
0
mock
control
miR-30e-3p
(%)
120
100
80
60
40
20
0
mock
control
miR-30e-3p
p < 0.001
(%)
120
100
80
60
40
20
0
mock
control
miR-30e-3p
C14orf126 expression
(relative to mock)
(%)
120
100
80
60
40
20
0
mock
control
miR-30e-3p
p < 0.001
p < 0.001
p < 0.001
p < 0.01
p < 0.01
(%)
120
100
80
60
40
20
0
mock
control
miR-30e-3p
PLS3 expression
(relative to mock)
(%)
120
100
80
60
40
20
0
mock
control
miR-30e-3p
(%)
120
100
80
60
40
20
0
mock
control
miR-30e-3p
ERGIC2 expression
(relative to mock)
(%)
120
100
80
60
40
20
0
mock
control
miR-30e-3p
(%)
120
100
80
60
40
20
0
mock
control
miR-30e-3p
HMGA2 expression
(relative to mock)
(%)
120
100
80
60
40
20
0
mock
control
miR-30e-3p
p < 0.001
p < 0.01
N.S.
p < 0.01
p < 0.05
p < 0.001
(%)
160
140
120
100
SERPINE1 expression
(relative to mock)
80
60
40
20
0
mock
control
miR-30e-3p
(%)
160
140
120
100
80
60
40
20
0
mock
control
miR-30e-3p
p < 0.001
p < 0.01
p < 0.001
(%)
120
100
80
60
40
20
0
mock
control
miR-30e-3p
PSMD10 expression
(relative to mock)
(%)
120
100
80
60
40
20
0
mock
control
miR-30e-3p
(%)
120
100
80
60
40
20
0
mock
control
miR-30e-3p
RALB expression
(relative to mock)
(%)
120
100
80
60
40
20
0
mock
control
miR-30e-3p
p < 0.001
p < 0.05
p < 0.001
p < 0.01
(%)
120
100
80
60
40
20
0
mock
control
miR-30e-3p
TMEM87B expression
(relative to mock)
(%)
120
100
80
60
40
20
0
mock
control
miR-30e-3p
(%)
120
100
80
60
40
20
0
mock
control
miR-30e-3p
(%)
120
100
80
60
40
20
0
mock
control
miR-30e-3p
SFXN1 expression
(relative to mock)
p < 0.01
p < 0.001
Supplemental Figure S5: Expression levels of 11 target genes in HNSCC cell lines (Sa3 and SAS)
All target genes were significantly downregulated in miR-30e-3p transfected samples except for HMGA2 in SAS. [N.S.: Not Significant]

## Slide 6
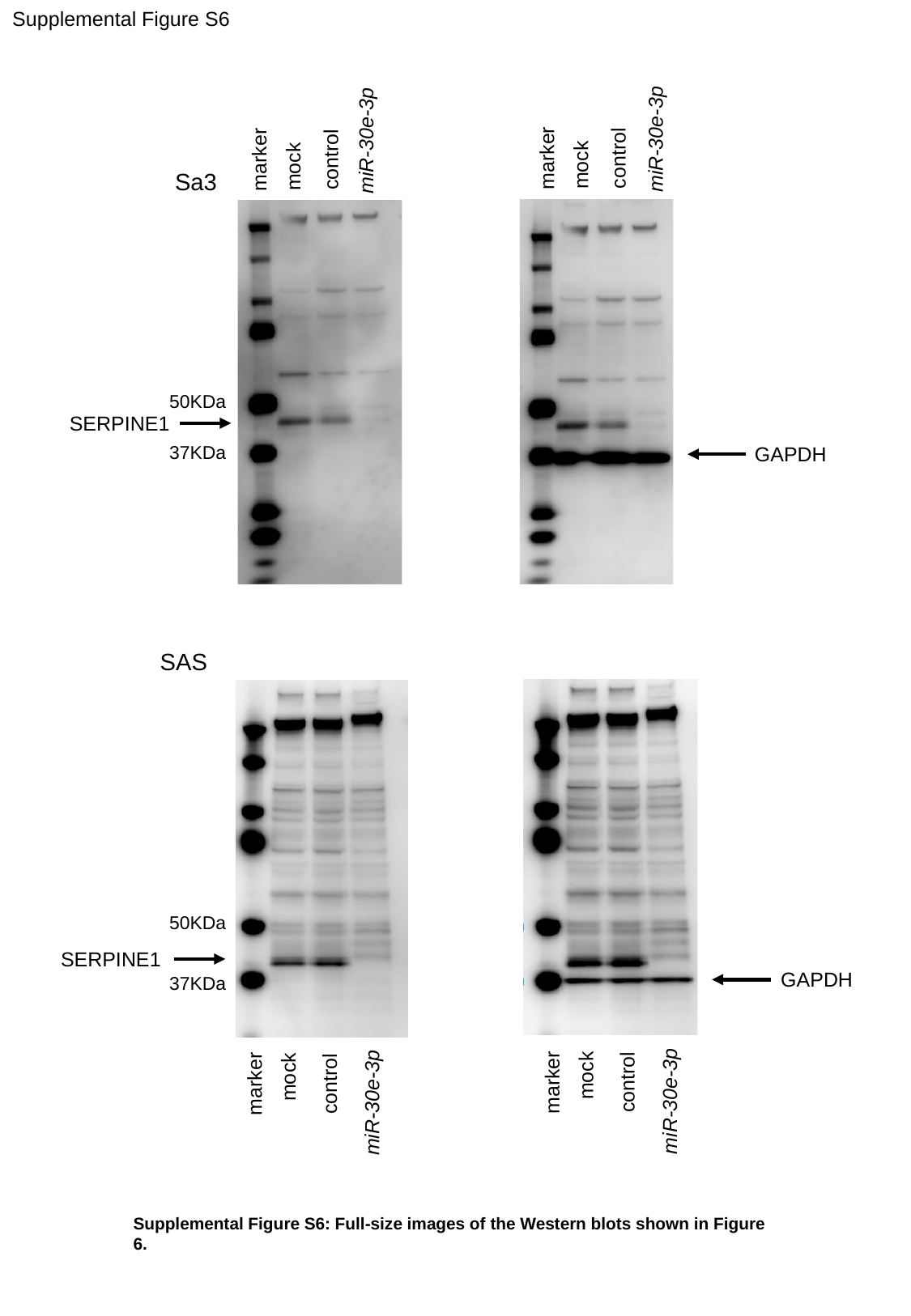

Supplemental Figure S6
miR-30e-3p
miR-30e-3p
marker
control
marker
control
mock
mock
Sa3
50KDa
SERPINE1
37KDa
GAPDH
SAS
50KDa
SERPINE1
GAPDH
37KDa
mock
mock
control
marker
control
marker
miR-30e-3p
miR-30e-3p
Supplemental Figure S6: Full-size images of the Western blots shown in Figure 6.

## Slide 7
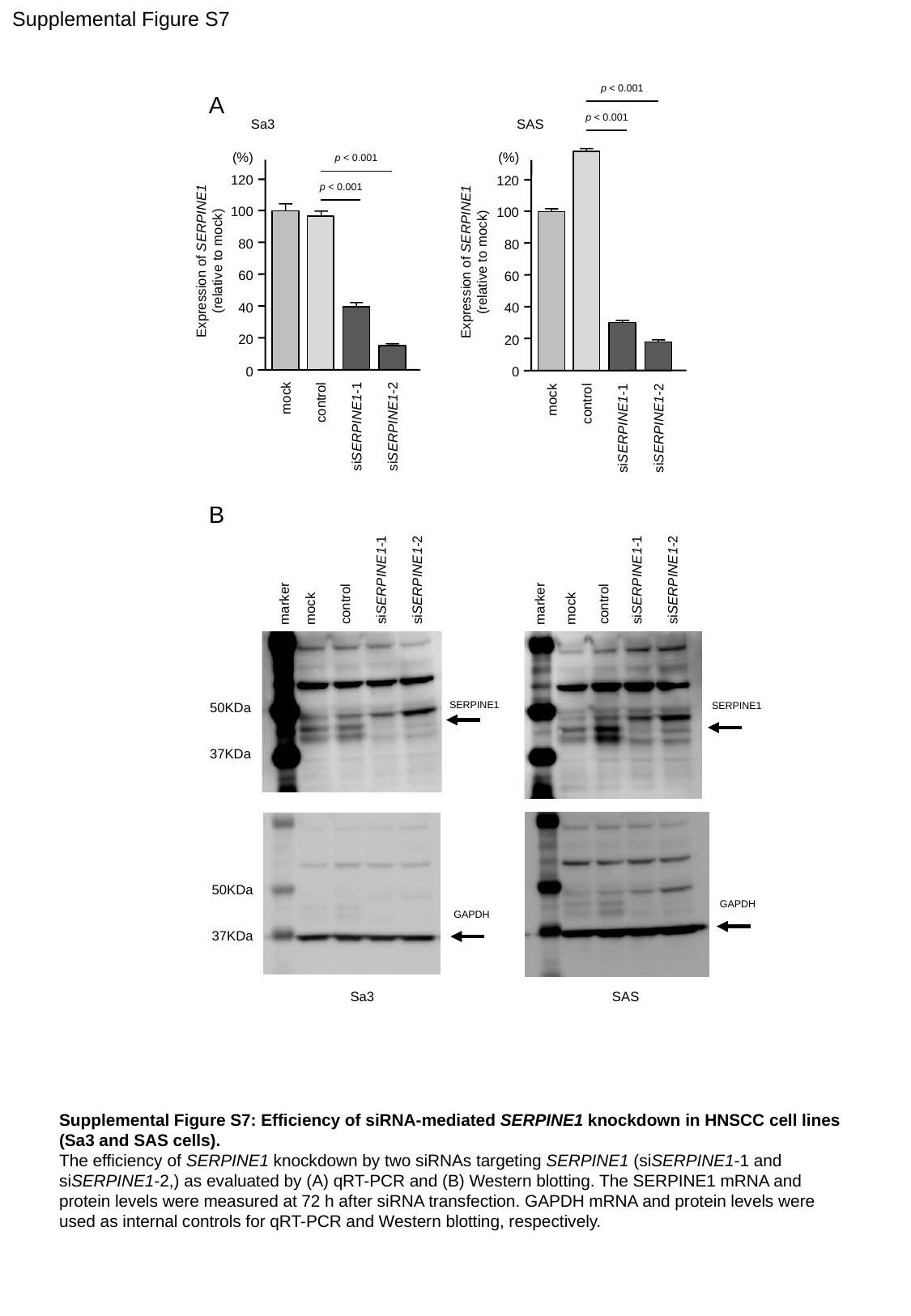

Supplemental Figure S7
p < 0.001
p < 0.001
SAS
(%)
120
100
80
Expression of SERPINE1
(relative to mock)
60
40
20
0
mock
control
siSERPINE1-1
siSERPINE1-2
A
Sa3
p < 0.001
p < 0.001
(%)
120
100
80
Expression of SERPINE1
(relative to mock)
60
40
20
0
mock
control
siSERPINE1-1
siSERPINE1-2
B
siSERPINE1-1
siSERPINE1-2
marker
control
mock
SERPINE1
50KDa
37KDa
50KDa
GAPDH
37KDa
siSERPINE1-1
siSERPINE1-2
marker
control
mock
SERPINE1
GAPDH
Sa3
SAS
Supplemental Figure S7: Efficiency of siRNA-mediated SERPINE1 knockdown in HNSCC cell lines (Sa3 and SAS cells).
The efficiency of SERPINE1 knockdown by two siRNAs targeting SERPINE1 (siSERPINE1-1 and siSERPINE1-2,) as evaluated by (A) qRT-PCR and (B) Western blotting. The SERPINE1 mRNA and protein levels were measured at 72 h after siRNA transfection. GAPDH mRNA and protein levels were used as internal controls for qRT-PCR and Western blotting, respectively.

## Slide 8
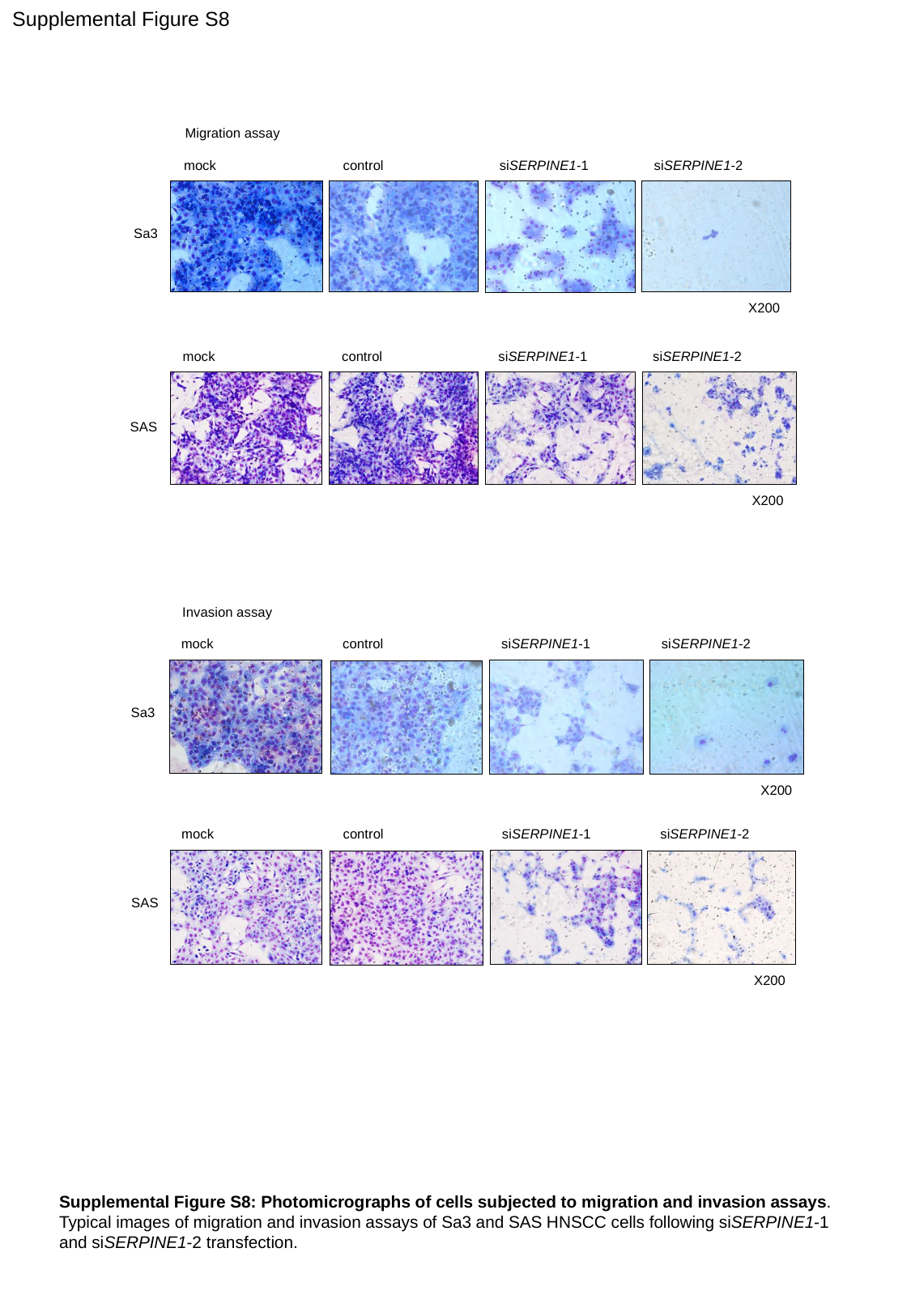

Supplemental Figure S8
Migration assay
mock
control
siSERPINE1-1
siSERPINE1-2
Sa3
X200
mock
control
siSERPINE1-1
siSERPINE1-2
SAS
X200
Invasion assay
mock
control
siSERPINE1-1
siSERPINE1-2
Sa3
X200
mock
control
siSERPINE1-1
siSERPINE1-2
SAS
X200
Supplemental Figure S8: Photomicrographs of cells subjected to migration and invasion assays.
Typical images of migration and invasion assays of Sa3 and SAS HNSCC cells following siSERPINE1-1 and siSERPINE1-2 transfection.
